# Improving emulsifying properties by high-voltage electrostatic field in emulsified pork batter as phosphate-replacement

**DOI:** 10.5713/ab.25.0384

**Published:** 2025-10-22

**Authors:** Yu-Tse Liu, Hui-Zhen Yan, Chao-Wei Huang, Thami-Wiseman Ndlandla, Fu-Yuan Cheng

**Affiliations:** 1 Department of Animal Science, National Chung Hsing University, Taichung City, Taiwan; 2 Department of Animal Science, National Pingtung University of Science and Technology, Pingtung, Taiwan; 3 Department of Tropical Agriculture and International Cooperation, National Pingtung University of Science and Technology, Pingtung, Taiwan

**Keywords:** Emulsification, High-voltage Electrostatic Field, Pork Batter, Scanning Electron Microscopy

## Abstract

**Objective:**

Phosphates are traditionally employed to improve emulsification and texture in meat products. In response to growing consumer demand for healthier, non-chemical alternatives, this study explored the potential of a non-thermal processing technology—high-voltage electrostatic field (HVEF)—to improve emulsification properties while preserving protein structures. Specifically, the objective was to evaluate the effects of HVEF treatment on the quality of phosphate-free emulsified pork batter.

**Methods:**

Fresh ham was cut and randomly allocated into four groups, including conventional refrigerator with 0.15% phosphate, phosphate (1) and phosphate-free without HVEF (0 kV/m) (2), and HVEF group, phosphate-free with low and high voltage −90 kV/m (3) and −150 kV/m (4) for 24 hours. Making the pork batter and analyzing the emulsifying activity index, emulsifying stability index, total expressible fluid and scanning electron microscope (SEM).

**Results:**

The results showed that the −150 kV/m HVEF group exhibited a significantly higher emulsifying activity index than Phosphate and 0 kV/m group (p<0.05), enhancing emulsification. After processing, the −90 kV/m HVEF group retained more moisture than phosphate and 0 kV/m groups (p<0.05). For total exudate loss (TEF) and fat loss, −150 kV/m group exhibited the highest TEF and fat loss, whereas the −90 kV/m group had lower TEF than 0 kV/m group, suggesting −90 kV/m HVEF improves emulsion stability. SEM revealed denser and smoother structures in phosphate and −90 kV/m groups.

**Conclusion:**

−90 kV/m HVEF enhances water retention and structural stability in emulsified pork batter, offering a viable phosphate alternative for high-quality meat products.

## INTRODUCTION

Emulsified meat products refer to products made from muscle tissue as the raw material, combined with fat, water, salt, spices, and other additives. These ingredients are emulsified into a meat batter using a high-speed mincer under low temperatures, followed by heating and cooking. Factors influencing emulsification include muscle proteins, fat, pH, additives, and processing methods [1]. Adjusting the ionic strength is one of the important functional roles of salts in meat products. Phosphates, with higher ionic strength than sodium chloride, help promote the dissolution and extraction of salt-soluble proteins. Adding phosphates to meat products enhances the pH, moving it away from the isoelectric point and increasing electrostatic repulsion forces. This separation between actin and myosin allows more water to be absorbed [2]. A higher pH benefits water retention and emulsification stability, the meat with a high pH exhibits better emulsifying properties and gel formation ability, while meat with a low pH results in a softer product texture [3]. Salt-soluble proteins extracted from normal meat with a pH of 6.5 possess a higher emulsifying capacity. Reducing the phosphate content in emulsified meat products leads to significant losses of water and fat, and the amount of phosphates added is positively correlated with the emulsification stability of the product [4].

In addition to chemical methods such as the use of food additives, recent studies have explored novel processing techniques, including ultrasound-assisted cooking [5], high hydrostatic pressure treatment [6], and high-pressure processing (HPP) [7], to investigate the effects of these techniques on meat product functional properties. HPP is a non-thermal food processing method primarily used to inactivate pathogenic and spoilage microorganisms, for processed meat products, HPP has been applied to reduce fat content [8], sodium levels [9], and nitrite usage [10]. Recent studies have also focused on HPP’s ability to remove or reduce phosphate levels in processed meat formulations. O’Flynn et al [7] found that applying HPP at 150 MPa for 5 minutes at ambient temperature reduced phosphate content in low-fat sausages to 0.25% without significantly altering their functionality.

High-voltage electrostatic field (HVEF) is another novel non-thermal processing technique that can reduce harmful changes in food quality and nutrition during processing while maintaining the physical and sensory properties of food [11]. The application of HVEF helps to increase the solubility of salt-soluble proteins in fish meat [12], indicating that HVEF has the potential to improve the emulsification of muscle proteins. Qi et al [13] studied the impact of electrostatic fields on the microbiological, nutritional, and physicochemical qualities of ready-to-eat fresh salmon stored at 6°C. The results showed that the application of HVEF did not significantly affect the amino acid or fatty acid composition in salmon meat. Additionally, HVEF reduced drip loss, protein degradation, and cohesiveness during storage. Thiobarbituric acid reacting substances (TBARS) results showed that HVEF promoted lipid oxidation during storage, followed by its inhibition over time. Jia et al [14] studied the role of HVEF in freezing pork tenderloin. The results indicated that HVEF-assisted freezing minimize the ice crystals formation compared to conventional freezing. Moreover, this method reduced protein denaturation, and after treatment with a 10 kV HVEF-assisted freezing, the meat color, pH, and water retention of the frozen meat were closer to the state of fresh pork tenderloin.

However, there is currently a lack of research on whether HVEF affects the water retention and emulsifying properties in emulsion meats. Therefore, this study aims to define the characteristics of emulsified product treated by HEVF technology and the underlying mechanism.

## MATERIALS AND METHODS

### Test materials, sample processing and high-voltage electrostatic field equipment

#### Materials and sample grouping

A total of three pork left fresh ham muscles (from commercial LYD [Landrace×Yorkshire× Duroc three-breed-crossing] pigs with an average carcass weight of approximately 100 kg) and pork back fat were obtained from a local slaughterhouse in Taiwan. Muscles were deboned 24 hours post-slaughter, and visible connective tissue and external fat were trimmed. The semimembranosus muscle (*M. semimembranosus*) was dissected and cut into 12 rectangular pieces (60×40×80 mm). The pork samples were randomly assigned to the Refrigerator group (6 pieces) and the HVEF group (−90 kV/m and −150 kV/m, 3 pieces each) for 24 hours of treatment (n = 1 ham per experiment, replicated 3 times). Subsequently, each group was processed into emulsified pork batter (the preparation method is shown in 2.1.2).

**Figure 1:**
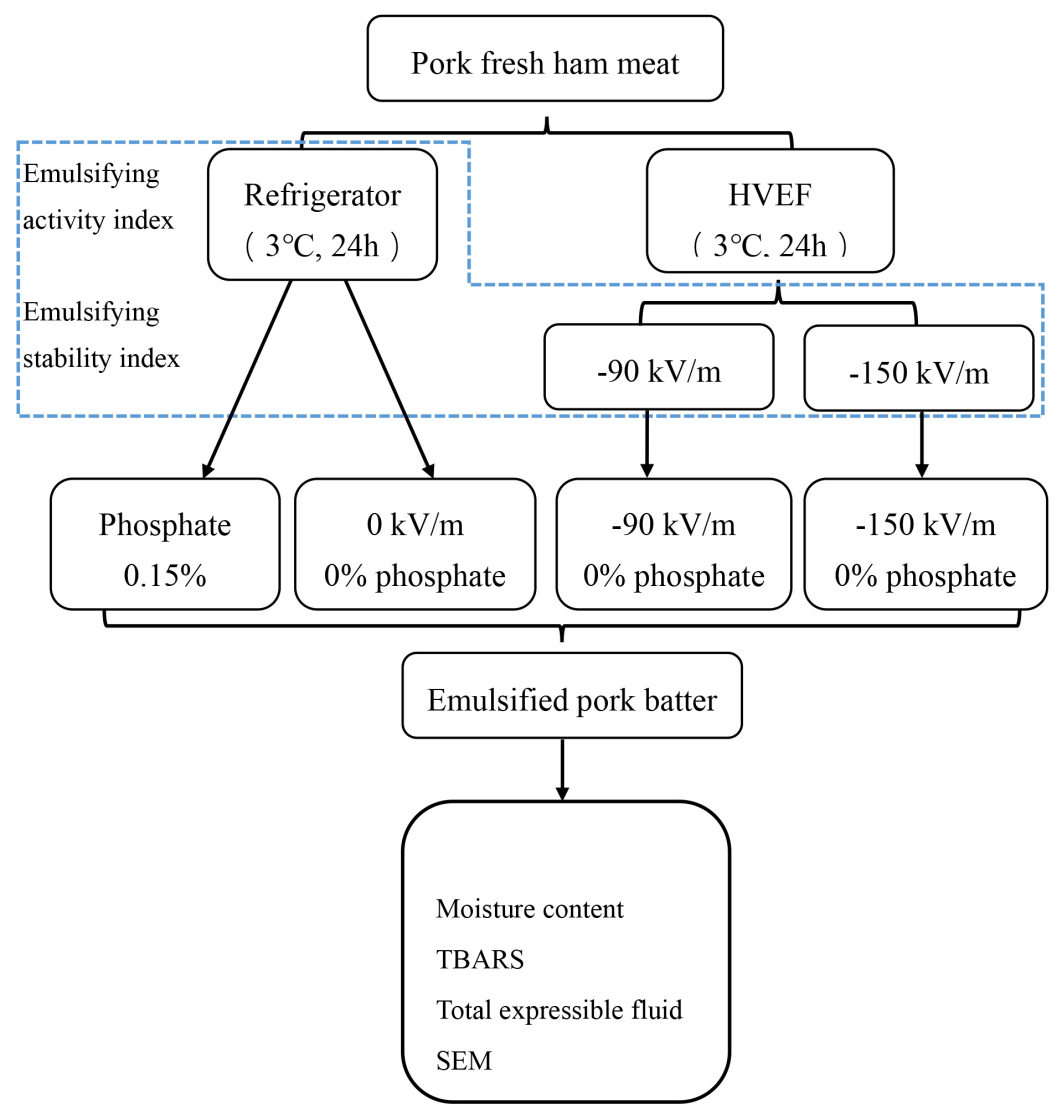
The experimental framework of each emulsified pork batter. HVEF, high-voltage electrostatic field; TBARS, thiobarbituric acid reacting substance; SEM, scanning electron microscope.

The refrigerator group was further divided into two formulations: one containing 0.15% phosphate (Phosphate) and the other without phosphate (0 kV/m). Both the −90 kV/m and −150 kV/m HVEF groups were phosphate-free (experimental framework is shown in Figure 1). The phosphate used in this study was a commercial blend (Chien-Yuan Food Technology) composed of sodium tripolyphosphate, potassium pyrophosphate, and sodium pyrophosphate. All experimental analysis were conducted in triplicate to minimize errors.

#### Emulsified pork batter preparation method

Emulsified pork batter was prepared according to Wei et al [15] with slight modifications. Before preparation, pork lean meat and back-fat were ground through a 6 mm and 3 mm plate separately using a meat grinder (PC-82/22; Mainca). The formula of emulsified pork batter was as follows: pork lean meat 70%, pork back fat 30%, ice 3%, salt 1.65%, composite phosphates 0.15% (only phosphate group used), sugar 2.5%, monosodium glutamate (MSG) 0.5%, and white pepper powder 0.2%.

The detailed processing procedures of emulsified pork batter were as follows: (1) The pork lean meat, salt, composite phosphates, and half of the ice were put into the cutter-emulsifier (R5-2V; Robot-Coupe) and beaten for 1 min (2,800 rpm). (2) Spices, and one fourth of the ice were added to the meat batter and beaten for 1 min (2,800 rpm). (3) The pork back fat and remaining ice were added to the meat batter and beaten for 30 s (2,800 rpm). The final temperature of meat batters was always below 10°C in all instances. After that, meat batters, which were prepared through beating processing, were shaped into pork batter ball with diameters of 35 mm (about 30 g of each pork batter, with each of the 4 groups producing approximately 20 pork batter balls per trial).

#### High-voltage electrostatic field equipment

**Figure 2:**
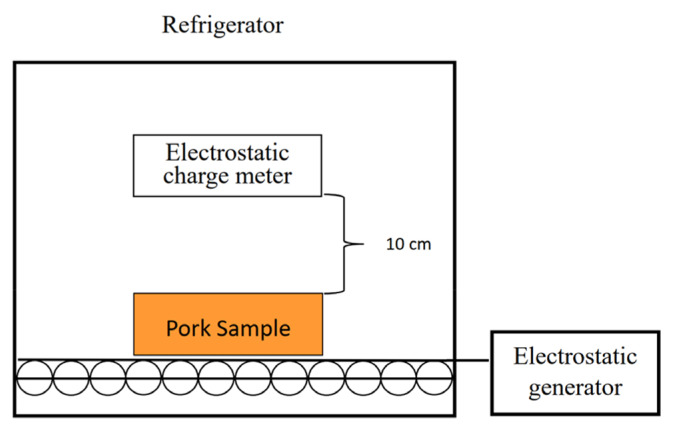
The structure of the refrigerator with high-voltage electrostatic field (HVEF).

The HVEF system used in this study is illustrated in Figure 2. An electrostatic generator (SC-PME 50; Cosmi) was installed outside the refrigerator and connected to two parallel plates inside the refrigerator, creating a continuous and uniform electrostatic field. Pork samples were placed between the plates and treated with different field intensities (−90 kV/m and −150 kV/m) for 24 hours.

### Emulsifying activity index and emulsifying stability index for myofibrillar protein isolate

#### Myofibrillar protein isolate

The method described by Jia et al [16] was employed for the analysis. Pork samples were minced using a blender (7010S; Waring). A 5 g portion of the minced pork sample was weighed and homogenized at 4°C with four volumes of phosphate buffer using a homogenizer (PT 10–35 GT; Kinematica) at 10,000 rpm for 30 seconds twice. The phosphate buffer was prepared with 0.1 M KCl, 2 mM MgCl_2_, 1 mM ethylene glycol tetraacetic acid (EGTA), and 1 mM phenylmethylsulfonyl fluoride (PMSF). After homogenization, the mixture was centrifuged at 2,000×g for 15 minutes using a centrifuge (Rotanta 460 R; Hettich), and the supernatant was discarded. The precipitate was washed twice with four volumes of the same phosphate buffer under the same centrifugation conditions. Subsequently, the precipitate was washed with four volumes of 0.1 M NaCl and centrifuged at 2,000×g for 15 minutes. The supernatant was discarded, and the precipitate was resuspended in eight volumes of 0.1 M NaCl, with the pH adjusted to 6.0. Finally, the protein content was determined using the DC protein assay (Bio-Rad).

#### Emulsifying activity index, emulsifying stability index

The modified method of Giménez et al [17] and Baridoet al [18] was employed for the analysis. A 15 mL sample of myofibrillar protein isolate (MPI) was transferred into a centrifuge tube, followed by the addition of 5 mL of soybean oil. The mixture was homogenized at 36,000×g for 1 minute using a homogenizer (PT 10–35 GT; Kinematica). Immediately after emulsion formation (A0), 50 μL of the emulsion was collected from 5 mm above the bottom of the centrifuge tube and dispersed in 5 mL of 0.1% sodium dodecyl sulfate (SDS) solution. This procedure was repeated at 10 minutes (A10). The absorbance at a wavelength of 500 nm was measured using a microplate reader (SPECTROstar Nano; BMG Labtech).


(1) $$EAl\left(m^{2}/g\right)=\frac{4.606\times A500}{C\times (1-\Phi )\times 10^{4}}\times \text{Dilutionmultiple}$$

C: Concentration of the sample before emulsification (mg/mL); A500: Absorbance value measured at 500 nm; Φ is the volume fraction of oil in the emulsion (v/v).


(2) ESI(%)=(A10/A0)×100

A0: Absorbance value of the emulsion measured at 0 minutes; A10: Absorbance value of the emulsion measured at 10 minutes.

### Moisture content

The method described by Rossa et al [19] was employed for moisture content determination. A 5 g sample of emulsified pork batter was placed on the sample pan of a moisture analyzer (ML-50; AND). Before measurement, the analyzer was pre-heated and operated under the recommended ambient conditions (5°C–40°C, ≤85% RH, no condensation). The standard mode was selected, in which the measurement conditions and termination values were automatically determined by the accuracy setting and the minimum scale display unit (0.1%). The analysis was considered complete when the drying rate fell below the preset termination value, and the final moisture content (%) was automatically recorded by the instrument.

### Total expressible fluid

The modified method of Hughes et al [20] was employed for analysis. A 25 g sample of emulsified pork batter was placed into a centrifuge tube and centrifuged at 2,700×g and 3°C for 1 minute using a centrifuge (Rotanta 460 R; Hettich). The sample was then heated in a water bath (BC-2D; Hipoint) at 70°C for 30 minutes, followed by centrifugation at 2,700×g for 3 minutes. The supernatant was poured into a pre-weighed crucible and dried in an oven (JA-72; Jorfai) at 100°C for 24 hours. The following calculations were performed:


(3) $$\begin{array}{l} \text{TEF}=(\text{Weight of centrifuge tube and sample})-\\ \left(\text{Weight of centrifuge tube and pellet}\right) \end{array}$$

(4) %TEF=(TEF/Sample weight)×100

(5) $$\begin{array}{l} \%\;\text{Fat}=([\text{Weight of crucible and dried supernatant}]-\\ {}[\text{Weight of empty crucible}]/\text{TEF})\times 100 \end{array}$$

### Thiobarbituric acid reacting substances test

The modified method from Puolanne et al [21]. A 5 g sample of emulsified pork batter was weighed and mixed with 10 mL of 20% trichloroacetic acid (TCA) and 10 mL of double-distilled water (DDW). The mixture was homogenized at 10,000 rpm for 30 seconds using a homogenizer (PT 10–35 GT; Kinematica). The homogenate was centrifuged at 1,200×g for 10 minutes using a centrifuge (Rotanta 460 R; Hettich) and then filtered through filter paper. A 2 mL aliquot of the filtrate was mixed with 2 mL of 0.02 M thiobarbituric acid (TBA) in a test tube. The mixture was heated in a water bath (BC-2D; Hipoint) at 95°C for 30 minutes. A blank was prepared using 1 mL of 20% TCA, 1 mL of DDW, and 2 mL of 0.02 M TBA. After heating, the samples were cooled under running water for 10 minutes. The absorbance at 530 nm was measured using a microplate reader (SPECTROstar Nano; BMG Labtech). The malondialdehyde (MDA) concentration was calculated using a standard curve.

### Scanning electron microscope

The procedure was adapted from the method described by He et al [22]. Emulsified pork batter (5 mm thick) was dehydrated using a freeze dryer (FD-5030; Panchum) and subsequently stored in a freezer (DW-86L628; Haier) at −80°C. A suitably sized portion of the sample, exposing both the surface and cross-section, was sputter-coated with gold using a sputter coater (E-1010; Hitachi). The sample was then observed and photographed using a scanning electron microscope (SEM; S3000N; Hitachi) at an accelerating voltage of 15 kV, and SEM images were acquired at a magnification of 200× to visualize the microstructure of the sample.

### Statistical analysis

Linear mixed model (LMM) analyses were used to analyze the results from the experiment, with the models fitted using SPSS (ver. 20.0; IBM) statistical software. Different treatment for pork was considered as fixed effects, and replicates (n = 3) were used as random effects. One-way analysis of variance (general linear model procedure) was conducted, and a Tukey-Kramer post-hoc test at a 95% confidence level (p< 0.05) was also applied using the same software to verify the differences among groups.

## RESULTS AND DISCUSSION

### Emulsification properties-emulsifying activity index and emulsifying stability index

The effects of HVEF treatment on the emulsifying activity index (EAI) and emulsifying stability index (ESI) of myofibrillar proteins from fresh pork fresh ham meat are presented in Table 1.

The EAI refers to the ability of proteins to adsorb at the oil-water interface after emulsification, preventing flocculation and aggregation, EAI estimates the relative surface coverage of protein on oil droplets in an emulsified state [23]. In this study, the −150 kV/m group exhibited a significantly higher EAI value (16.87 m^2^/g) compared to the refrigerator group (12.11 m^2^/g) and the −90 kV/m group (12.68 m^2^/g) (p<0.05). Huang et al [24] noted that larger protein particles tend to aggregate, potentially resulting in oversized proteins that require longer migration times and are less aligned at the oil-water interface. This reduced flexibility during adsorption at the interface leads to lower EAI values [23]. Furthermore, Xu and Liu [25] highlighted the importance of surface charge in influencing EAI values, as higher surface charges increase electrostatic repulsion between proteins, reducing aggregation tendencies. Amiri et al [26] observed that myofibrillar proteins extracted from beef treated with 10 kV HVEF during thawing had smaller protein particles compared to a control group thawed at 20°C. They attributed this to corona discharge generated by HVEF, which ionized the air and increased the net surface charge of myofibrillar proteins. Charged ions also altered non-covalent interactions, causing structural changes in proteins, such as chain unfolding, increased surface area, and reduced particle size [26]. Based on these findings, the present study suggests that −150 kV/m HVEF treatment reduced the particle size of myofibrillar proteins in fresh pork, increased surface charge, and enhanced electrostatic repulsion between proteins. This prevented aggregation at the oil-water interface, thereby improving emulsifying activity.

The ESI assesses the ability of proteins to maintain emulsion stability by measuring the rate of destabilization over time. It reflects the stability of fat globules achieved through protein-protein and protein-fat interactions. A decrease in ESI is often attributed to protein denaturation [27]. In this study, the Refrigerator group exhibited a significantly higher ESI value (82.62%) compared to the −150 kV/m group (73.05%), with the −90 kV/m group (73.69%) falling in between. Similarly, Huang et al [24] found that HVEF treatment decreased the ESI value of myofibrillar proteins extracted from catfish. Myofibrillar protein variation is a key factor influencing emulsification capacity and is affected by storage and processing conditions. Sun et al [28] reported that HVEF treatment at 4 kV increased sulfhydryl group content in duck myofibrillar proteins, indicating protein unfolding and exposure of internal groups. This improved protein surface hydrophobicity and enhanced the gel elasticity and viscosity of myofibrillar proteins. However, Amiri et al [26] found that HVEF treatment at 10 kV reduced sulfhydryl content in beef myofibrillar proteins, attributing this to ozone formation in the HVEF environment, which oxidized hydroxyl groups and formed disulfide bonds, altering protein structure. In the Refrigerator group of this study, hydrophobic residues were likely hidden within the folded structure of native proteins, maintaining protein structure through hydrophobic interactions [28]. Collectively, it is hypothesized that the intense corona discharge and ozone release during HVEF treatment induced myofibrillar protein denaturation, resulting in decreased ESI values.

The pH values of emulsified pork batters under different HVEF treatments were previously established in our related study [29]. Briefly, the −150 kV/m and 0 kV/m groups exhibited significantly lower pH values than the phosphate and −90 kV/m groups (p < 0.05). The higher pH levels observed in the phosphate-added and −90 kV/m groups are critical, as they enhance electrostatic repulsion between protein molecules and increase the distance between protein chains, thereby facilitating water retention through muscle fiber swelling and protein activation [30]. Consequently, this elevated pH directly contributes to superior water-holding capacity, yield stress, and elasticity in the emulsified products. Conversely, a decrease in pH may result from an increase in solute concentration and protein denaturation, leading to the release of hydrogen ions and subsequent water loss [31].

### Effect of high-voltage electrostatic field treatment on moisture content of emulsified pork batter

**Figure 3:**
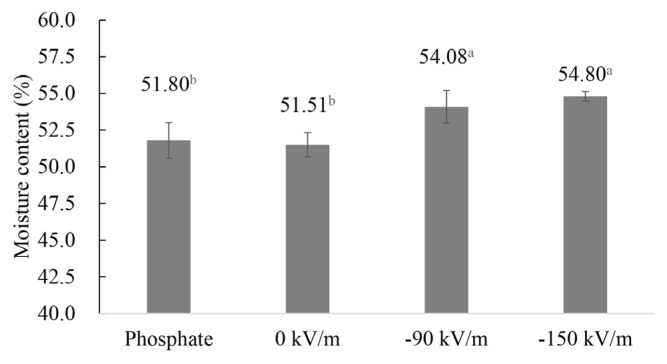
Effect of HVEF treatment on the moisture content of emulsified pork batter (n = 3). Phosphate: 0.15% phosphate; 0 kV/m: 0% phosphate; −90 kV/m: 0% phosphate with −90 kV/m HVEF; −150 kV/m: 0% phosphate with −150 kV/m HVEF. Error bars show the standard deviation from the mean. ^a,b^ Different superscripts represent significant differences (p<0.05). HVEF, high-voltage electrostatic field.

The changes in moisture content of emulsified pork batters prepared from fresh pork fresh ham meat treated with HVEF are shown in Figure 3. The −90 kV/m group (54.08%) and the −150 kV/m group (54.80%) exhibited significantly higher moisture content compared to the phosphate group (51.80%) and the 0 kV/m group (51.51%). These results indicate that HVEF treatment can retain moisture in the emulsified product. Xu et al [32] studied pork *M. Longissimus thoracis et lumborum* (LTL) stored under different modes of 12 kV HVEF for 4, 8, 12, and 16 days. They found that groups continuously treated with HVEF maintained significantly higher moisture content during storage, mitigating moisture loss over time. This effect may be attributed to the interaction of charged particles and hydrogen bonds with water molecules, enhancing their binding to proteins, and reducing moisture loss. Ko et al [33] treated tilapia fillets with 300, 600, and 900 kV/m HVEF and conducted an 8-day storage test. Their results showed that the initial moisture content of the fish was 78.6%, which dropped to 74.0% after 8 days in the control group, whereas the HVEF-treated groups maintained moisture content above 76%. The study suggested that HVEF delays protein denaturation, effectively preserving moisture in fish fillets. Additionally, previous research indicated that the charges generated by electrostatic fields can transform disordered water molecular structures into ordered dendritic structures. HVEF may introduce these charges, enabling water molecules in fresh meat to move more orderly when exposed to the electrostatic field, thereby reducing moisture loss [34].

### Effect of high-voltage electrostatic field treatment on total expressible fluid and fat of emulsified pork batter

The changes in total expressible fluid (TEF) and fat loss of emulsified pork batters prepared from fresh pork fresh ham meat treated with HVEF are shown in Table 2. TEF and fat loss are key parameters for evaluating the ability of myofibrillar proteins to retain water and fat in meat products, which are closely related to product yield [7]. Higher TEF and fat loss indicate lower emulsion stability. The −150 kV/m group exhibited the highest TEF among all groups (3.00%). Notably, the −90 kV/m group (0.15%) had lower TEF compared to the 0 kV/m group (1.37%), neither of these two meat products had added phosphates. Regarding fat loss, the −150 kV/m group (25.00%) and the 0 kV/m group (18.51%) were significantly higher than the phosphate group (3.25%) and the −90 kV/m group (3.26%). Phosphates can dissociate the actomyosin complex, releasing myosin to function as a natural emulsifier. Additionally, phosphates can extract more myofibrillar proteins, providing a stable protein matrix that binds water and fat more effectively [4]. This may explain the lower TEF of the phosphate group, indicating better emulsion stability. In the 0 kV/m group without added phosphates, water and fat loss were higher than the phosphate group and −90 kV/m group. Jia et al [14] noted that HVEF can delay the denaturation and oxidation of myofibrillar proteins, helping maintain protein structure and their ability to bind water and fat. However, excessive voltage may lead to protein structure damage. It is speculated that the high voltage applied in the −150 kV/m group caused extensive corona discharge during HVEF treatment, ionizing the air and generating ozone. This ozone likely reacted with the secondary and tertiary structures of proteins, altering helices, sheets, and coils, thereby reducing their ability to bind water and fat.

### Effect of high-voltage electrostatic field treatment on thiobarbituric acid reacting substances value of emulsified pork batter

**Figure 4:**
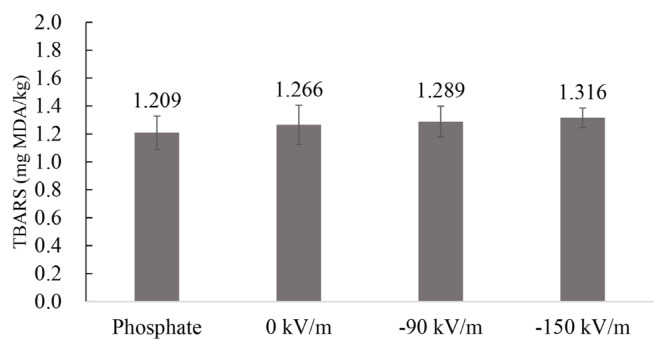
Effect of HVEF treatment on TBARS of emulsified pork batter (n = 3). Phosphate: 0.15% phosphate; 0 kV/m: 0% phosphate; −90 kV/m: 0% phosphate with −90 kV/m HVEF; −150 kV/m: 0% phosphate with −150 kV/m HVEF. Error bars show the standard deviation from the mean. TBARS, two-thiobarbituric acid reacting substance; MDA, malondialdehyde; HVEF, high-voltage electrostatic field.

The changes in TBARS values of emulsified pork batters prepared from fresh pork fresh ham meat treated with HVEF are shown in Figure 4. No significant differences were observed among the groups in TBARS values. However, a slight upward trend was noted in the two HVEF-treated groups, with the −150 kV/m group showing the highest TBARS value (1.316 mg MDA/kg) and the phosphate group the lowest (1.209 mg MDA/kg). Kılıç et al [35] demonstrated that adding phosphates to minced chicken and beef reduces lipid oxidation, thereby lowering TBARS values. Molins et al [36] found that thermal hydrolysis of sodium tripolyphosphate and sodium pyrophosphate during cooking increases orthophosphate concentrations. The rise in orthophosphate levels can reduce phosphatase activity, thereby mitigating oxidation. Additionally, the chelating properties of phosphates provide antioxidant effects, as phosphates complexed with metal ions can inhibit lipid oxidation. Otherwise, metal ions might catalyze oxidation of proteins like hemoglobin and lipids like phospholipids. Therefore, adding phosphates to products helps prevent the development of rancid off-flavors [37]. Amiri et al [26] investigated the effects of different electrode configurations in an HVEF system on thawing beef tenderloin and observed that TBARS values significantly increased with the number of pin electrodes. They also found that lipid oxidation during storage was exacerbated as the number of electrodes increased. After 10 days of refrigeration, the group with the highest number of pin electrodes showed TBARS values 3.15 times higher than the control group. During air ionization in the HVEF system, oxygen molecules are dissociated into oxygen atoms, producing ozone. An increase in electrode numbers elevates current flow, leading to higher ozone production. High ozone concentrations can oxidize sample surfaces, as ozone is a strong oxidizing agent that induces lipid oxidation in oxidation-sensitive foods, thereby impairing flavor. In this study, 24 hours of HVEF treatment on fresh pork fresh ham meat had no negative impact on lipid oxidation in the samples.

### Effect of high-voltage electrostatic field treatment on surface and internal microstructure of emulsified pork batter (scanning electron microscope)

**Figure 5:**
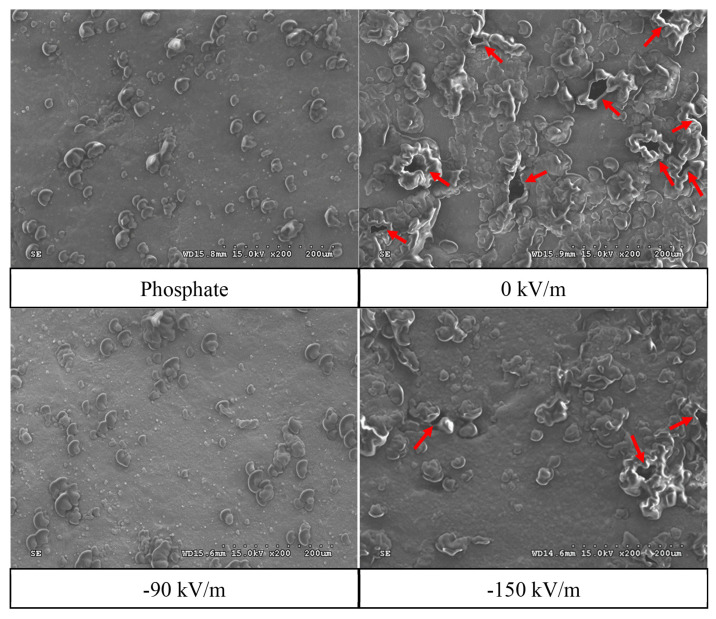
Effect of HVEF treatment on the surface microstructure of emulsified pork batter (200×). Phosphate: 0.15% phosphate; 0 kV/m: 0% phosphate; −90 kV/m: 0% phosphate with −90 kV/m HVEF; −150 kV/m: 0% phosphate with −150 kV/m HVEF. Arrow indicates more and larger pores on the surface. HVEF, high-voltage electrostatic field.

The surface microstructure of emulsified pork batter prepared from fresh pork fresh ham meat samples treated with HVEF was observed via SEM, as shown in Figure 5. The SEM images revealed that the phosphate group and the −90 kV/m group exhibited a denser and smoother emulsified batter structure with smaller and fewer pores. In contrast, the 0 kV/m group and the −150 kV/m group showed more and larger pores on the surface of the emulsified pork batter. This indicates that moisture loss from the meat matrix led to poorer emulsion stability. The SEM observations were consistent with the results of total liquid and fat loss, where the 0 kV/m and −150 kV/m groups experienced more severe moisture and fat loss.

**Figure 6:**
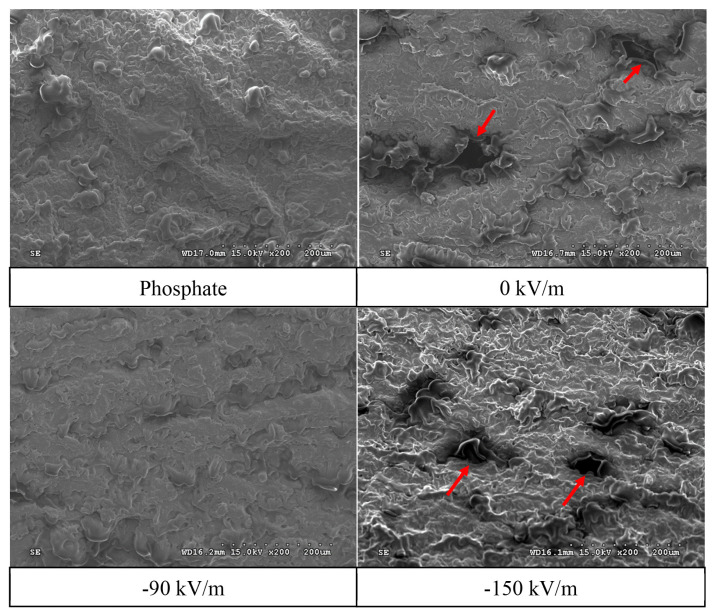
Effect of HVEF treatment on the internal microstructure of emulsified pork batter (200×). Phosphate: 0.15% phosphate; 0 kV/m: 0% phosphate; −90 kV/m: 0% phosphate with −90 kV/m HVEF; −150 kV/m: 0% phosphate with −150 kV/m HVEF. Arrow indicates rougher textures with more pores on the internal. HVEF, high-voltage electrostatic field.

The internal microstructure of emulsified pork batter prepared from fresh pork fresh ham meat samples treated with HVEF was also observed via SEM, as shown in Figure 6. Similar to the surface microstructure, the phosphate group and the −90 kV/m group exhibited smooth and stable structures, while the 0 kV/m and −150 kV/m groups had rougher textures with more pores, which could negatively affect the texture of the final product.

Pores on the surface of emulsified meat batter play a crucial role in the emulsion system. Larger pores tend to result in greater fat loss during heating, leading to poorer emulsion stability. Moreover, a denser surface structure indicates a thinner protein layer encapsulating fat globule [38]. He et al [22] found that the cross-section of emulsified chicken batter displayed a rough surface with irregular and large pores, likely caused by rapid moisture loss during heating and drying, leading to irregular and unsealed cavities in the internal structure. Zhuang et al [39] also noted that pores in the protein gel structure act as water channels. Consequently, larger pores facilitate water leakage from the protein network structure. The emulsion characteristics also showed a decrease in ESI values for the −90 kV/m and −150 kV/m groups. SEM images suggested that HVEF at −90 kV/m induced slight denaturation of myofibrillar proteins by exposing internal protein groups. On the other hand, the −150 kV/m group may have experienced intense corona discharge and ozone generation due to excessive voltage [40], accelerating protein denaturation. Although the higher EAI value indicated an increased surface coverage of proteins on oil droplets, the excessive protein denaturation compromised stability over time, resulting in the significantly lowest ESI value.

It should be noted that although we attempted to monitor ozone concentration during HVEF treatment, the recorded values were highly variable due to the low-temperature environment and air circulation in the refrigerator. As integrating an additional ozone sensor was technically challenging, reliable ozone data could not be obtained, which represents a limitation of this study. This type of emulsified pork batter is often used in heat-processed products. However, in this study, we aimed to specifically investigate the effects of HVEF treatment on pork before heating, focusing on emulsifying properties, emulsifying stability, and microstructure. In the future, we plan to conduct further experiments on heat-processed products, including analyses of color, shear force, fracturability, texture profile analysis, and sensory evaluation, which will be discussed in our next study.

## CONCLUSOIN

HVEF treatment significantly affects the physicochemical properties of phosphate-free emulsified pork batter. The −90 kV/m group exhibited a pH level comparable to that of the phosphate group, indicating improved water retention. Moisture content analysis showed higher retention in the −90 kV/m group, with lower TEF than the 0 kV/m group, suggesting better emulsion stability. Lipid oxidation (TBARS) showed no significant differences among groups, though it was slightly higher in HVEF-treated samples, especially the −150 kV/m group. SEM analysis revealed that the −90 kV/m group had fewer pores, indicating a denser structure, while the −150 kV/m and 0 kV/m groups had larger pores and lower stability. Overall, −90 kV/m HVEF treatment effectively enhances water retention and structural stability in emulsified pork batter, demonstrating potential as a phosphate alternative.

## Figures and Tables

**Table 1:** Effect of HVEF treatment on emulsifying activity index and emulsifying stability index of myofibrillar protein isolates from pork samples (n = 3)

	Refrigerator	−90 kV/m	−150 kV/m	SEM
EAI (m^2^/g)	12.11^b^	12.68^b^	16.87^a^	0.43
ESI (%)	82.62^a^	73.69^ab^	73.05^b^	2.11

Refrigerator: regular refrigerator 24 hours; −90 kV/m: −90 kV/m HVEF 24 hours; −150 kV/m: −150 kV/m HVEF 24 hours.

a,b Different superscripts in the same row represent significant differences (p<0.05).

HVEF, high-voltage electrostatic field; SEM, standard error of the mean; EAI, emulsifying activity index; ESI, emulsifying stability index.

**Table 2:** Effect of HVEF treatment on total expressible fluid and fat of emulsified pork batter (n = 3)

	Phosphate	0 kV/m	−90 kV/m	−150 kV/m	SEM
TEF (%)	0.15^b^	1.37^ab^	0.15^b^	3.00^a^	0.43
Fat (%)	3.25^b^	18.51^a^	3.26^b^	25.00^a^	2.55

Phosphate: 0.15% phosphate; 0 kV/m: 0% phosphate; −90 kV/m: 0% phosphate with −90 kV/m HVEF; −150 kV/m: 0% phosphate with −150 kV/m HVEF.

a,b Different superscripts in the same row represent significant differences (p<0.05).

HVEF, high-voltage electrostatic field; SEM, standard error of the mean; TEF, total expressible fluid.

## Data Availability

Upon reasonable request, the datasets of this study can be available from the corresponding author.

## SUPPLEMENTARY MATERIAL

**Supplement 1**. Experiment device of high-voltage electrostatic field (HVEF) system.

**Supplement 2**. Analysis of different conditions on pH value, TBARS, TSP, DL, and CL of HVEF-treated pork hind leg samples (preliminary experiment data).

**Supplement 3**. Hematoxylin and eosin staining of tissue.
