# Supplementary material for: Improving emulsifying properties by high-voltage electrostatic field in emulsified pork batter as phosphate-replacement
Source: Anim Biosci. 2025 Oct 22;39(3):250384. doi: 10.5713/ab.25.0384 (PMC12963747; doi:10.5713/ab.25.0384)
Supplement: Supplementary file 1 [file ab-25-0384-Supplementary-1.pdf]

# Supplementary materials

The experiment device of high-voltage electrostatic field (HVEF) system is illustrated in Supplement 1. Consisted of an electrostatic generator (SC-PME 50, Cosmi, New Taipei, Taiwan) and connected to strip parallel plate (60cm X 100cm) inside the refrigerator. Pork samples were placed on strip parallel plate and an electrostatic charge meter (SK-H050, Keyence, Osaka, Japan) positioned 10 cm from the samples was used to measure and adjust the field intensity to –90 kV/m and –150 kV/m, individually. A continuous and uniform electrostatic field was maintained for 24 hours.

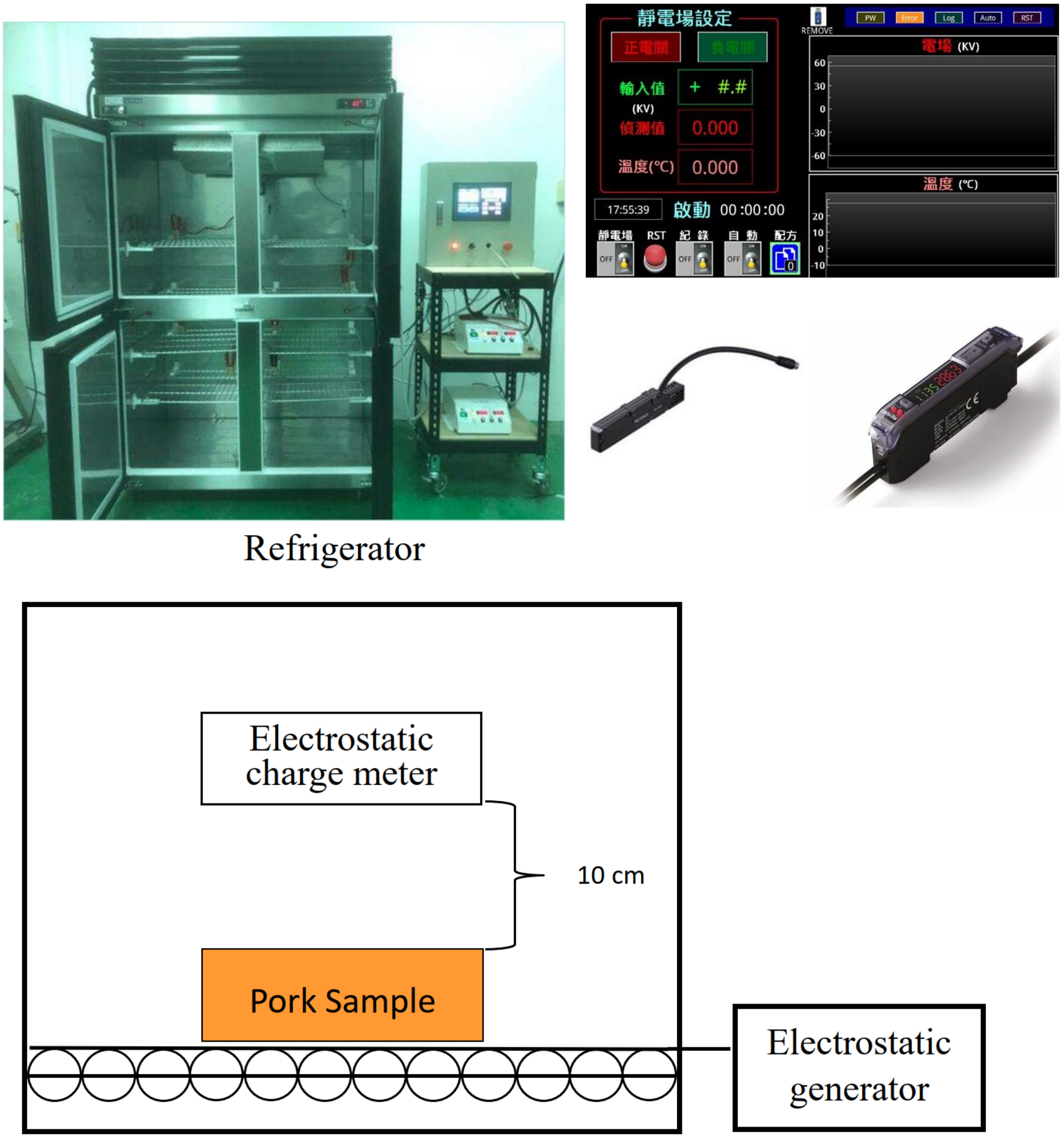


**Supplement 1.** Experiment device of high-voltage electrostatic field (HVEF) system.
