# Supplementary material for: Improving emulsifying properties by high-voltage electrostatic field in emulsified pork batter as phosphate-replacement
Source: Anim Biosci. 2025 Oct 22;39(3):250384. doi: 10.5713/ab.25.0384 (PMC12963747; doi:10.5713/ab.25.0384)
Supplement: Supplementary file 2 [file ab-25-0384-Supplementary-2,3.pdf]

**Supplement 2.** Analysis of different conditions on pH value, TBARS, TSP, DL, and CL of HVEF-treated pork hind leg samples (preliminary experiment data)

| Traits | Control | -60kV/m | -90kV/m | -120kV/m | -150kV/m | SEM |
|---|---|---|---|---|---|---|
| pH | $6.18^{a}$ | $5.82^{ab}$ | $5.79^{ab}$ | $5.91^{ab}$ | $6.10^{a}$ | 0.10 |
| TBARS[1] (mg MDA/kg) | $0.70^{d}$ | $1.01^{bc}$ | $1.30^{ab}$ | $0.75^{cd}$ | $1.29^{ab}$ | 0.10 |
| TSP[2] (g/100g meat) | $10.52^{ab}$ | $10.74^{a}$ | $9.19^{c}$ | $9.95^{b}$ | $8.09^{d}$ | 0.12 |
| DL[3] (%) | - | $1.85^{ab}$ | $1.91^{ab}$ | $0.95^{b}$ | $0.98^{b}$ | 0.18 |
| CL[4] (%) | $20.32^{d}$ | $23.73^{bc}$ | $22.36^{cd}$ | $26.03^{ab}$ | $24.54^{ab}$ | 0.79 |

[1]TBARS, Thiobarbituric Acid Reactive Substances

[2]TSP, Total soluble-protein solubility

[3]DL, Drip loss

[4]CL, Cooking loss

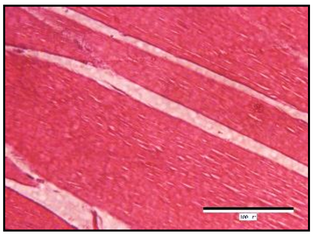

**(A) Control**

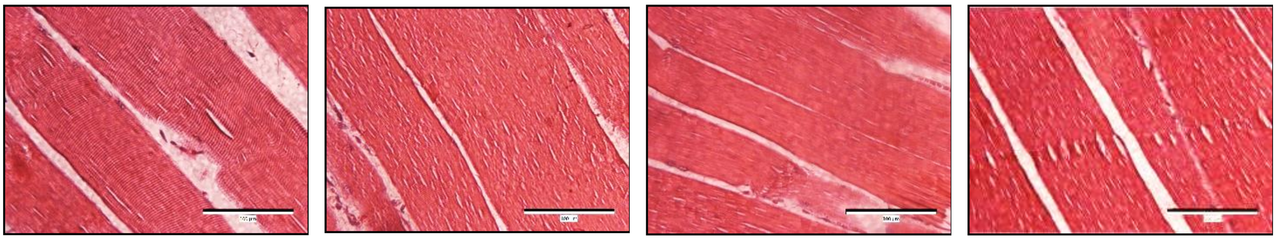

**(B) -60** **(C) -90** **(D) -120** **(E) -150**

**Supplement 3.** Hematoxylin and eosin staining of tissue. From left to right are the A: Control, B: -60kV/m, C: -90kV/m, D: -120kV/m, E: -150kV/m, Transverse section of myofibril. Scale bar = 100 μm. (preliminary experiment data)
